# Patterns of resource utilization and cost for postmenopausal women with hormone-receptor–positive, human epidermal growth factor receptor-2–negative advanced breast cancer in Europe

**DOI:** 10.1186/s12885-015-1762-3

**Published:** 2015-10-24

**Authors:** Guy Jerusalem, Patrick Neven, Nina Marinsek, Jie Zhang, Ravi Degun, Giancarlo Benelli, Stephen Saletan, Jean-François Ricci, Fabrice Andre

**Affiliations:** 1 Centre Hospitalier Universitaire Sart Tilman Liège and Liège University, Domaine Universitaire du Sart Tilman, B35, 4000 Liège, Belgium; 2 University Hospitals Leuven, Herestraat 49, box 7003, B-3000 Leuven, Belgium; 3 Navigant Consulting, Inc, Woolgate Exchange, 25 Basinghall Street, EC2V 5HA London, UK; 4 Novartis Pharmaceuticals Corporation, One Health Plaza, East Hanover, NJ 07936-1080 USA; 5 Novartis Farma S.p.A., Largo Umberto Boccioni 1, I-21040 Origgio, VA Italy; 6 Wellmera AG, Basel, Switzerland; 7 Institut Gustave Roussy, 39 Rue C. Desmoulins, 94805 Villejuif, France

**Keywords:** Advanced breast cancer, Direct costs, Europe, Resource utilization, Work productivity

## Abstract

**Background:**

Healthcare resource utilization in breast cancer varies by disease characteristics and treatment choices. However, lack of clarity in guidelines can result in varied interpretation and heterogeneous treatment management and costs. In Europe, the extent of this variability is unclear. Therefore, evaluation of chemotherapy use and costs versus hormone therapy across Europe is needed.

**Methods:**

This retrospective chart review (*N =* 355) examined primarily direct costs for chemotherapy versus hormone therapy in postmenopausal women with hormone-receptor–positive (HR+), human epidermal growth factor receptor-2–negative (HER2–) advanced breast cancer across 5 European countries (France, Germany, The Netherlands, Belgium, and Sweden).

**Results:**

Total direct costs across the first 3 treatment lines were approximately €10 000 to €14 000 lower for an additional line of hormone therapy-based treatment versus switching to chemotherapy-based treatment. Direct cost difference between chemotherapy-based and hormone therapy-based regimens was approximately €1900 to €2500 per month. Chemotherapy-based regimens were associated with increased resource utilization (managing side effects; concomitant targeted therapy use; and increased frequencies of hospitalizations, provider visits, and monitoring tests). The proportion of patients taking sick leave doubled after switching from hormone therapy to chemotherapy.

**Conclusions:**

These results suggest chemotherapy is associated with increased direct costs and potentially with increased indirect costs (lower productivity of working patients) versus hormone therapy in HR+, HER2– advanced breast cancer.

**Electronic supplementary material:**

The online version of this article (doi:10.1186/s12885-015-1762-3) contains supplementary material, which is available to authorized users.

## Background

Breast cancer is one of the most commonly diagnosed cancers in women, with an estimated 463 819 new cases diagnosed in Europe in 2012 [1]. The economic burden of this disease is also high; across the European Union, breast cancer generated the highest estimated healthcare costs (6 billion Euros/year) and accounted for 13 % of the total healthcare costs for cancer [2, 3]. However, healthcare resource utilization in breast cancer varies by disease stage and treatment choice [4]. In advanced breast cancer (ABC), hormone therapy and chemotherapy are treatment options that have (to some extent) guideline-specific recommendations regarding initiation of use [5–10].

Hormone therapy is recommended as adjuvant therapy and is viewed as standard of care for hormone-receptor–positive (HR^+^) ABC [6–9]. The value of adjuvant chemotherapy in this setting is unclear [11], and most guidelines recommend sequential endocrine therapies except in patients with proof of hormone resistance or symptomatic visceral disease [8, 9, 12]. However, guidelines for the sequence and preferred number of hormone therapy lines that can be used before switching to chemotherapy in ABC—outside of medical necessity—are not always clear [8, 9, 12]. This lack of clarity can result in varied interpretation of guidelines and can lead to heterogeneous treatment management and costs.

Use of chemotherapy in HR^+^ ABC is associated with extensive healthcare costs in the United States (US) [13–16]; evaluations of chemotherapy costs for HR^+^ ABC in Europe have not been reported. For example, a US study of 1266 women with HR^+^ ABC reported that treatment costs for the year following initiation of chemotherapy were $32 083 higher than the 1-year treatment costs before chemotherapy [14]. Furthermore, a recent evaluation of total direct costs in the US for treating ABC reported that the monthly per-patient direct cost was lowest with systemic hormone therapy ($5303) compared with chemotherapy ($13 261) [13]. The cost of chemotherapy in the US can be primarily attributed to costs other than the drug itself (25 % drug cost and 75 % nondrug costs such as infusion administration and hospitalizations or emergency room visits related to drug) [16]. Because European treatment patterns may vary from those in the US, similar evaluation of chemotherapy use and costs versus hormone therapy across Europe is needed.

This chart review evaluates the resource utilization and direct cost implications of chemotherapy versus hormone therapy based on actual physician-reported treatments from adjuvant therapy to completion of 3 or more lines of therapy in the advanced setting in postmenopausal women diagnosed with HR^+^, human epidermal growth factor receptor-2–negative (HER2^−^) ABC from 2008 through 2012 in France, Germany, The Netherlands, Belgium, and Sweden.

## Methods

### Study design

This retrospective chart review was conducted from April to June 2012 by physicians or healthcare providers (HCPs) in the areas of gynecology and medical or clinical oncology who treat ABC. The participating medical professionals were recruited from across France, Germany, The Netherlands, Belgium, and Sweden to complete a questionnaire based on their patient charts. Selection of the medical professionals was based on years of clinical practice postresidency or postfellowship (≥5 but ≤35 years), time spent treating patients (≥60 %), and the number of patients with breast cancer for whom they were responsible for systemic treatment decisions in the year preceding the study (≥50 but ≤1000 patients). Medical professionals were contacted via email to assess their interest in participation (based on a database of breast cancer oncologists and record of previous participation in such research), and a follow-up phone call was made to discuss details of the research when requested by the potential participant. All participating physicians electronically signed a consent form before entering data. Data collected in the questionnaire were from anonymous patient charts, and the study was compliant with both European and individual country regulations. Ethics approval was deemed not applicable for this study because it was done under market research regulations through a physician panel (fully double blinded physician list) and only collected fully anonymous patient chart information without any patient identifiers or ability to follow-up with physicians. Online patient record forms did not collect any data that would (or could reasonably) lead to the patient being identified (no name, address, postal code, date of birth, etc.). No patient or physician identifier is recorded in the database, and only aggregated data were shared with the sponsor. The survey methodology was compliant with guidelines from a number of market research and pharma associations. A list of authorities this survey methodology was compliant with at the time of survey administration is available in Additional file 1: Table S1.

The study objective was to understand the treatment patterns and quantify resource utilization of HR^+^, HER2^−^ ABC, with the overall aim of describing the costs as patients progress in the ABC setting.

### Chart inclusion criteria

The key inclusion criteria for charts reviewed were postmenopausal women with HR^+^, HER2^−^ ABC, defined as metastatic or locally advanced breast cancer not amenable to curative treatment by surgery or radiotherapy; living or deceased patients with recurring or *de novo* diagnosis were eligible, and the diagnosis of ABC had to be made in 2008 through 2012. For a chart to be eligible, the patients had to have progressed on at least 1 hormone therapy line in the adjuvant or advanced disease setting (could be administered with chemotherapy or targeted therapy) and had to have completed at least 1 chemotherapy line (minimum 2 cycles) in the ABC setting after hormone therapy.

### Data collection

Data collected in the questionnaire included patient demographics and disease state and characteristics at the initiation of each treatment line, together with information on any/all metastases, and all comorbidities (please see Additional file 2: Figure S1 for a copy of the full questionnaire). Maintenance therapy was considered a separate treatment line. Treatment details were requested at each line, including agent dose, duration, and administration route. Data were also collected on patient performance status and side effects of chemotherapy and any complementary treatments to alleviate those effects. Reasons for switching to the next line of treatment were also collected. The information collected on resource utilization at each treatment line included number of physician visits (office and outpatient), hospitalizations (by diagnosis-related group codes, where available) and duration of stay, any additional treatments or HCP visits, disease monitoring information (type, frequency, location, and medical professional responsible), and working status.

### Statistical analysis

Information from the questionnaires was summarized by line of therapy. As a result, the charts were divided into 3 cohorts by key treatment algorithms based on sequence of hormone therapy and chemotherapy. Following adjuvant therapy, hormone therapy-sensitive disease was defined as relapsed >1 year after adjuvant therapy and hormone therapy-refractory disease as relapsed during adjuvant therapy or within 1 year after adjuvant therapy. A subgroup analysis of patients eligible for hormone therapy at second line was defined by response to hormone therapy of ≥6 months in the previous line of therapy, no significant metastasis progression, and/or no visceral crisis or brain metastases. Direct and indirect costs due to treatments were summarized by descriptive statistics. The unit costs by month and by treatment line for drugs, monitoring, hospitalizations, HCP visits, and palliative care were calculated for each patient chart based on the cost assumptions for each country from 2012 in Euros (Table 1). No adjustments for inflation were made, as the charts included were from a relatively short 4-year time span (2008–2012) during which the inflation rate in the European Union was ~8.1 %, which is not considered to be a significant enough change to impact the resource utilization frequency/distribution [17]. The sums of each unit cost for all patient charts in each cohort were then averaged. Costs were not analyzed per country because of low patient numbers. The Web-based Mann-Whitney U test [18] (Wilcoxon rank-sum test) was used for group cost comparisons, with 2-sided *p* values. Analyses were performed using Microsoft Excel.

**Table 1:** Unit cost data by country in Euros

	France^a^	Germany^a^	Netherlands^a^	Belgium^a^	Sweden^a^
Healthcare Provider Visit					
Oncologist	45.00	50.65	72.00	54.98	126.47
General practitioner	23.00	35.75	28.00	23.67	73.03
Physiotherapist	30.00	20.00	35.00	25.00	36.63
Dietician	61.07	20.00	27.00	30.00	50.00
Psychotherapist	37.00	33.30	77.00	50.00	100.00
Outpatient (ambulatory care)	150.00	157.61	150.00	120.00	285.93
Day-care hospitalization	295.51	200.00	251.00	350.00	529.43
Home nurse visits	40.00	36.16	35.00	40.00	68.03
Palliative care (outpatient)	40.00	36.16	35.00	40.00	68.03
Hospitalization (inpatient)	3204.00	3317.61	2931.18	2534.00	4007.79
Palliative care (inpatient)	6346.00^b^	1339.98^c^	3057.17^d^	3500.00^b^	5981.83^b^
Diagnostic/Monitoring Test					
CBC	8.37	9.10	8.74	8.15	8.74
Blood chemistry panel	38.37	39.95	32.00	32.62	41.74
Blood tumor markers	16.70	8.70	9.00	25.00	20.74
Creatinine (urine)	2.07	1.80	2.07	1.75	1.74
Hematuria (urine)	2.35	1.25	2.35	1.75	1.74
Bicarbonates (urine)	2.35	1.25	2.35	1.75	1.74
Mammography	66.42	62.96	83.48	41.89	77.65
Bone X-ray	47.88	87.97	106.63	60.00	70.71
Chest X-ray	42.56	50.00	77.74	50.08	89.00
DXA	39.96	30.90	31.50	47.00	57.96
PET-CT	1034.00	1100.00	1454.80	1000.00	1390.80
CT scan	313.10	225.71	218.04	200.00	298.44
MRI	365.11	558.28	377.29	250.00	700.00
Bone scintigraphy	180.44	226.20	189.19	200.00	250.00
Liver ultrasound	56.70	61.58	38.20	ND	123.29
Primary tumor biopsy	176.80	193.93	205.97	261.41	274.57
Metastases biopsy	176.80	193.93	205.97	261.41	274.57
Electrocardiogram	13.52	19.80	39.40	19.80	22.26

Abbreviations: *CBC* complete blood count, *CT* computed tomography, *DXA* dual-energy x-ray absorptiometry, *MRI* magnetic resonance imaging, *ND* no data, *PET-CT* positron emission tomography–computed tomography

^a^Cost per order for each country: France, August 2012; Germany, September 2012; The Netherlands, June 2012; Belgium, November 2012; Sweden, November 2012

^b^One-time cost

^c^Per admission

^d^Mean stay, 8 days

## Results

### Physician base

Ninety-four physicians contributed 399 eligible patient charts (Table 2). There were similar numbers of physicians from each of the 5 European countries; however, physicians from France contributed ~25 % of the total number of charts. The majority of physicians reported a medical oncology specialty (62 %), whereas 23 % reported clinical oncology and 15 % reported gynecology (in some countries gynecologists treat patients with ABC) as their speciality. The majority of physicians reported that they treat patients at a teaching hospital (47 %) or general hospital (32 %), and 63 % of physicians treated 50 to 200 patients in the year preceding the study.

**Table 2:** Evidence base for chart review

Country	Charts, *n*	Participating physicians, *n*	Gynecologists, %^a^
France	105	21	—
Germany	79	21	48
The Netherlands	68	19	—
Belgium (Flemish region)	84	17	24
Sweden	63	16	—
TOTAL	399	94	15

^a^Percentage of participating physicians who were gynecologists

### Patient base

**Fig. 1:**
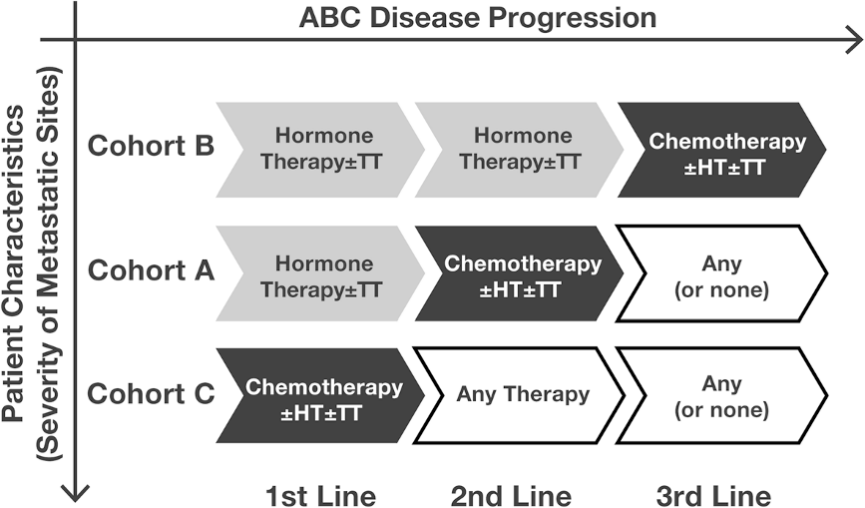
Flow diagram showing the methodology for comparison of resource utilization in the three cohorts. Abbreviations: ABC, advanced breast cancer; HT, hormone therapy; TT, targeted therapy

Treatment patterns allowed the division of 355 patients with ABC into 3 cohorts: cohort A (hormone therapy first line, chemotherapy second line, and any treatment third line), cohort B (hormone therapy first and second lines, and chemotherapy third line), and cohort C (chemotherapy first line, and any treatment second and third lines) (Fig. 1, Table 3). In general, patient demographics and disease characteristics were similar across the 3 cohorts (Table 4). However, patients in cohort C were more likely to have a family history of breast and/or ovarian cancer and to present with liver and brain metastases at ABC diagnosis. The remaining 44 patient charts were excluded from the analysis because they did not meet the criteria for these 3 cohorts: 36 patients received only 1 therapy line in ABC and 8 patients received hormone therapy for 3 lines of treatment before switching to chemotherapy.

**Table 3:** Patient cohorts recorded in the chart review

	Cohort A (*n* = 218) HT 1st line, CT 2nd line, Any Trx 3rd line	Cohort B (*n* = 26) HT 1st line, HT 2nd line, CT 3rd line	Cohort C (*n* = 111) CT 1st line, Any Trx 2nd line Any Trx 3rd line
Average duration of 3 therapy lines, months	20.9	22.9	19.7
Breast cancer history at ABC diagnosis, *n* (%)			
Recurring during adjuvant therapy	37 (17)	3 (12)	30 (27)
Recurring ≤1 year after adjuvant therapy	14 (6)	2 (8)	21 (19)
Recurring >1 year after adjuvant therapy	66 (30)	13 (50)	38 (34)
*De novo* ABC	101 (46)	8 (31)	22 (20)
Adjuvant drug therapies, *n* (%)			
Any	102 (47)	17 (65)	86 (77)
None	116 (53)	9 (35)	25 (23)
First-line ABC setting, *n* (%)			
Hormone therapy	218 (100)	26 (100)	32 (29)
Chemotherapy	0	0	111 (100)
Targeted therapy	7 (3)	0	24 (22)
Second-line ABC setting, *n* (%)			
Hormone therapy	18 (8)	26 (100)	59 (53)
Chemotherapy	218 (100)	0	65 (59)
Targeted therapy	45 (21)	1 (4)	13 (12)
Third-line ABC setting, *n* (%)			
Hormone therapy	31 (14)	1 (4)	12 (11)
Chemotherapy	39 (18)	26 (100)	31 (28)
Targeted therapy	10 (5)	2 (8)	9 (8)
None	149 (68)	0	73 (66)

Abbreviation: *ABC* advanced breast cancer, *CT* chemotherapy, *HT* hormone therapy, *Trx* treatment

**Table 4:** Patient demographics and disease characteristics

	Cohort A (*n* = 218)	Cohort B (*n* = 26)	Cohort C (*n* = 111)	Overall (*n* = 355)
Median age, years	64	61	61	63
Family history, breast/ovarian cancer, *n* (%)	35 (16)	3 (12)	29 (26)	67 (19)
ECOG performance status, *n* (%)				
0–1	168 (77)	26 (100)	94 (85)	288 (81)
2–4	44 (20)	0	17 (15)	61 (17)
Missing	6 (3)	0	0	6 (2)
AJCC stage grouping, *n* (%)				
IIIA	41 (19)	4 (15)	16 (14)	61 (17)
IV	146 (67)	22 (85)	70 (63)	238 (67)
PgR-positive status, *n* (%)^a^	155 (71)	19 (73)	69 (62)	243 (68)
Tumor proliferation, *n* (%)^a^				
Ki-67 status <20 %	26 (12)	2 (8)	20 (18)	48 (14)
Grade 3	100 (46)	11 (42)	53 (48)	164 (46)
Metastatic site, *n* (%)				
Brain	6 (3)	0	13 (12)	19 (5)
Lung	72 (33)	6 (23)	36 (32)	114 (32)
Liver	61 (28)	3 (12)	52 (47)	116 (33)
Bone	118 (54)	22 (85)	60 (54)	200 (56)

Abbreviations: *AJCC* American Joint Committee on Cancer, *ECOG* Eastern Cooperative Oncology Group, *PgR* progesterone receptor

^a^May reflect baseline characteristics before advanced breast cancer (ABC) diagnosis when biopsy was not conducted at ABC diagnosis

The majority of patient charts (62 %) fit the treatment pattern for cohort A, and in this cohort the largest percentage of patients was diagnosed with *de novo* ABC (46 %). Approximately 50 % of these patients were diagnosed with hormone-sensitive recurrent disease. Cohort C consisted of 31 % of the patient charts. Patients in this cohort were primarily diagnosed with recurrent disease and were evenly split between hormone-refractory and hormone-sensitive. Cohort B was excluded from further analyses because of the low patient numbers (*n* = 26).

The majority of patients in each cohort received adjuvant treatment: 47 % in cohort A and 77 % in cohort C. Overall, hormone therapy was the most common adjuvant therapy across cohorts (79 % and 93 %, respectively). However, chemotherapy use and targeted therapy use were higher in cohort C (81 % and 15 %, respectively) compared with cohort A (56 % and 5 %, respectively). A small group of patients in each cohort received anti-HER2 therapy (ie, lapatinib or trastuzumab) despite being recorded as having HR^+^, HER2^−^ disease. Anti-HER2 therapy was prescribed for 13 patients (23 prescriptions) in cohort A and 17 patients (21 prescriptions) in cohort B. These anti-HER2 prescriptions accounted for approximately 10 % of the overall treatment costs reported here.

### Direct costs

The overall cost differences between hormone therapy and chemotherapy across all 3 cohorts combined indicates that hormone therapy in the first or first and second line is associated with lower costs compared with chemotherapy in the same treatment settings (Table 5). Furthermore, substantial direct cost differences were identified for patients whose disease progressed during or within 1 year of adjuvant hormone therapy (hormone therapy-refractory) as well as for patients whose disease progressed more than 1 year after adjuvant endocrine therapy or who presented with *de novo* advanced breast cancer (hormone therapy-eligible).

**Table 5:** Cost differences between chemotherapy-based and hormone therapy-based therapy options based on hormone therapy sensitivity^a^

		Total Cohort Analysis	Subgroup analysis	
Patient group description		All patients (*n* = 355)	HT-refractory^b^ in 1st line (*n* = 107)	HT-eligible^c^ in 2nd line (*n* = 248)
Total cost difference (over 3 lines of therapy)^d^	HT instead of CTx in 1st line	€14 362	€7300	NA
	HT instead of CTx in 2nd line	€10 368	NA	€10 300
Cost difference (for 1 line of therapy)^d^	1st line HT vs 2nd line CTx	€9879 (~€1900/mo)	€7550 (~€1650/mo)	NA
	1st line HT vs 1st line CTx	€15 167 (~€2500/mo)	€13 850 (~€2350/mo)	NA
	2nd line HT vs 2nd line CTx	€8201 (~€1700/mo)	NA	€8550 (~€1700/mo)

Abbreviations: *ABC* advanced breast cancer, *CTx* chemotherapy, *HT* hormone therapy, *mo* month, *NA* not applicable

^a^Numbers in the table reflect cost savings with HT versus CTx

^b^Hormone refractory indicated recurrence within 1 year of adjuvant HT

^c^Hormone eligible indicates *de novo* (no adjuvant therapy) or recurrent disease ≥1 year following adjuvant HT

^d^Costs analysis was based on unit cost assumptions for each country, which were then averaged

**Fig. 2:**
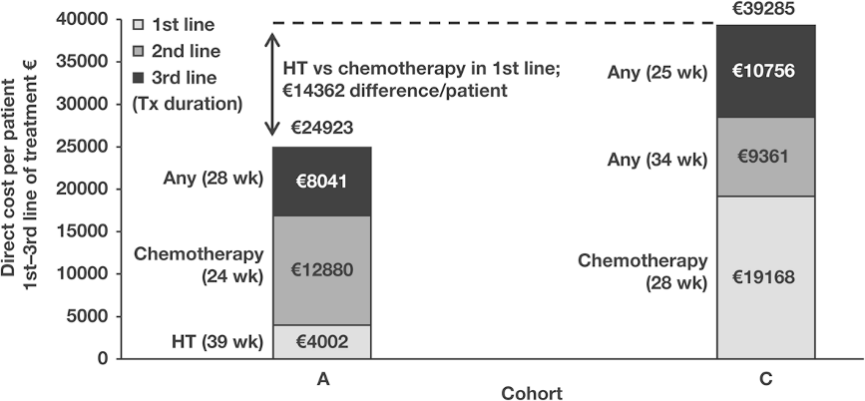
Direct overall costs by patient cohort. The costs in cohort A were significantly lower than the costs in cohort C (*p* < 0.001, Mann-Whitney U test). Average treatment durations by type and line of therapy in each cohort are shown in parentheses. Abbreviations: HT, hormone therapy; Tx, treatment; wk, week(s)

**Fig. 3:**
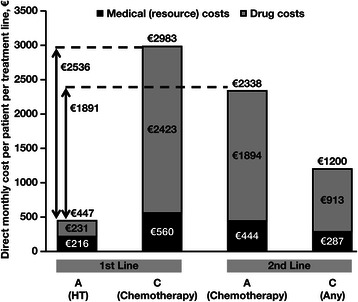
Direct average monthly costs by patient cohort and line of treatment. Costs in cohort A 1st line (HT) were significantly lower compared with costs in cohort A 2nd line (chemotherapy) and cohort C (any therapy; *p* < 0.001 for both comparisons, Mann-Whitney U test). Abbreviation: HT, hormone therapy

Over all 3 treatment lines, total direct costs were significantly lower in cohort A, which received hormone therapy first line, compared with cohort C (*p* < 0.001, Mann-Whitney U test), which received chemotherapy first line (Fig. 2). Chemotherapy as first-line treatment (cohort C) increased overall costs by €14 362 compared with first-line hormone therapy (cohort A), despite longer average duration of overall treatment in cohort A versus cohort C (20.9 vs 19.7 months, respectively). A subgroup analysis of patients who were eligible for hormone therapy as second-line treatment (*n* = 248) showed that use of chemotherapy as second-line treatment instead of hormone therapy increased overall costs by €10 302. Because duration of treatment varied across lines and types of therapy within each cohort, monthly direct costs were also examined, and they were found to have the same pattern as overall costs. In first-line therapy, the monthly direct costs for chemotherapy were €2536 higher versus hormone therapy (*p* < 0.001, Mann-Whitney U test) (Fig. 3). In second-line therapy, the monthly direct costs for chemotherapy were €1891 higher compared with hormone therapy in the first line (*p* < 0.001, Mann-Whitney U test). A subgroup analysis of patients who were eligible for hormone therapy as second-line treatment showed that use of chemotherapy as second-line treatment instead of hormone therapy increased monthly costs by a similar amount (€1705).

### Drivers of increased direct costs for chemotherapy

**Fig. 4:**
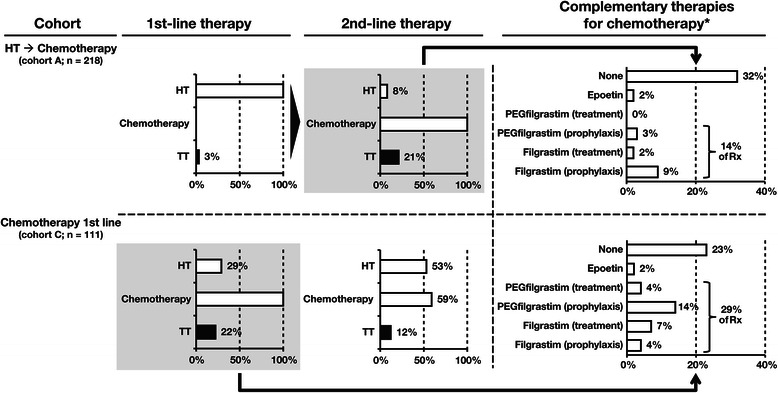
Overall use of concomitant targeted therapies and complementary treatments to manage chemotherapy side effects. Abbreviations: HT, hormone therapy; Rx, prescriptions; TT, targeted therapy. *Data presented for first line of chemotherapy treatment in each cohort

**Fig. 5:**
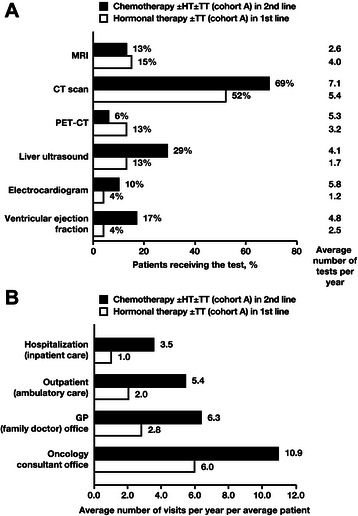
Cohort A: hormone therapy versus chemotherapy regimens for (**a**) monitoring tests and (**b**) healthcare resource utilization. Cohort A received HT 1st line followed by chemotherapy 2nd line and any treatment 3rd line. Abbreviations: CT, computed tomography; GP, general practitioner; HT, hormone therapy; MRI, magnetic resonance imaging; PET, positron emission tomography; TT, targeted therapy

Most of the increased cost for chemotherapy was due to the drug cost itself (Table 6), and there was increased use of other therapies with chemotherapy. Compared with first-line hormone therapy (cohort A), first-line chemotherapy (cohort C) was associated with approximately 7-fold increased use of concomitant targeted therapies and with increased use of complementary treatments to manage chemotherapy side effects (Fig. 4). Bevacizumab was the most commonly used targeted therapy, followed by trastuzumab and lapatinib. Increased resource utilization resulted in approximately double the resource costs for chemotherapy compared with hormone therapy (Table 6). Moreover, the analysis of healthcare resource utilization in cohort A showed that second-line chemotherapy was associated with increased frequencies of hospitalizations, ambulatory care and HCP visits, and a subset of monitoring tests compared with first-line hormone therapy (Fig. 5). A similar trend was observed for chemotherapy in the other cohorts (data not shown).

**Table 6:** Costs contributing to monthly direct cost for chemotherapy-based and hormone therapy-based treatment

	Average monthly costs (median monthly costs) and contribution percentages^a^						
	Contributor	Hormone therapy 1st line, Cohort A (*n* = 218)		Chemotherapy 1st line, Cohort C (*n* = 111)		Chemotherapy 2nd line, Cohort A (*n* = 218)	
Drug^b^	Hormone therapy	€159	35.5 %	€57	1.9 %	€23	1.0 %
		(€78)		(€0)		(€0)	
	Chemotherapy	NA	NA	€1145	38.4 %	€873	37.3 %
				(€942)		(€515)	
	Targeted therapy	€72	16.1 %	€897	30.1 %	€863	36.9 %
		(€0)		(€0)		(€0)	
	Chemotherapy complementary treatment	NA	NA	€325	10.9 %	€135	5.8 %
				(€0)		(€0)	
	Total	€231	51.6 %	€2424	81.2 %	€1894	81.0 %
Resource	Diagnostic/monitoring	€135	30.2 %	€298	10.0 %	€212	9.1 %
		(€114)		(€225)		(€157)	
	Hospitalization^c^	€12	2.6 %	€75	2.5 %	€54	2.3 %
		(€0)		(€0)		(€0)	
	Physician visits^d^	€64	14.4 %	€163	5.5 %	€155	6.6 %
		(€19)		(€113)		(€109)	
	Other HCP visits^e^	€5	1.0 %	€15	0.5 %	€7	0.3 %
		(€0)		(€5)		(€3)	
	Palliative care^f^	€0.3	0.1 %	€10	0.3 %	€15	0.6 %
		(€0)		(€0)		(€0)	
	Total	€216.3	48.4 %	€561	18.8 %	€443	19.0 %
Average monthly cost		€447		€2983		€2338	

Abbreviations: *HCP* healthcare professional, *NA* not applicable

^a^Costs and percentages are rounded to the nearest Euro (where possible) and 10th place, respectively, which may lead to slight differences in some totals. Note that median costs may be markedly different from the average costs. For example, if a large number of patients in a group had no hospitalizations while a small number of patients in the group had hospitalizations, the median costs may be zero even though the average costs are high

^b^Includes drug cost only to avoid double-counting; administration costs are included in the medical costs below under the assumption that all outpatient visits are recorded

^c^Includes daycare inpatient treatments (e.g., drug administration or monitoring) and longer hospitalization

^d^Oncology consultant office, general practitioner, ambulatory care, and nurse home visit

^e^Physiotherapist, dietician, and psychotherapist visits

^f^Includes daycare and hospitalization for palliative care

### Indirect costs

Indirect cost increases were attributed to the lower productivity of working patients: at ABC diagnosis 43 % of patients in cohort A and 56 % of patients in cohort C were working full or part time. In cohort A, there was a greater increase in sick leave during chemotherapy use compared with hormone therapy, which lowered the productivity of working-age patients and would result in indirect cost increases (Table 7). This increase in sick leave during chemotherapy versus hormone therapy was also observed across cohorts during the same line of treatment, with a notable increase during second-line treatment for cohort A (41 % for chemotherapy) compared with first-line treatment (19 % for hormone therapy). Additionally, in cohort A the increase in sick leave was accompanied by a substantial corresponding decrease in the proportion of patients working during second-line treatment (10 % for chemotherapy) compared with first-line treatment (32 % for hormone therapy). In contrast, only a small decrease in the proportion of working patients was observed between first line chemotherapy (26 %) and any second line therapy (22 %) in Cohort C. Overall, the working categories of retired (normal and early) and unemployed remained relatively stable over the course of the 3 treatments in both cohorts.

**Table 7:** Working status^a^ during ABC treatment for patients <65 years of age

	Patients, *n* (%)			
	Full-time work	Part-time work	Sick leave	Retired early
Cohort A				
ABC diagnosis (*n* = 109)	26 (24)	21 (19)	6 (6)	7 (6)
1st-line hormone therapy (*n* = 109)	13 (12)	22 (20)	21 (19)	8 (7)
2nd-line chemotherapy (*n* = 109)	2 (2)	9 (8)	45 (41)	10 (9)
3rd-line any therapy (*n* = 39)	1 (3)	2 (5)	16 (41)	5 (13)
Cohort C				
ABC diagnosis (*n* = 70)	26 (37)	13 (19)	5 (7)	4 (6)
1st-line chemotherapy (*n* = 70)	6 (9)	12 (17)	29 (41)	4 (6)
2nd-line any therapy (*n* = 70)	8 (11)	8 (11)	29 (41)	6 (9)
3rd-line any therapy (*n* = 27)	2 (7)	1 (4)	14 (52)	2 (7)

Abbreviation: *ABC* advanced breast cancer

^a^The percentages of patients do not add to 100 % because the working status categories of voluntary work, unemployed, retired, and unknown did not have appreciable changes within the cohorts over time and are not presented

## Discussion

Several treatment options are provided by international guidelines for European patients with ABC at each point in disease progression. However, physicians in individual countries may have limited treatment choices that are guided by country-specific restrictions, separate guidelines, or required procedures. Furthermore, specific guidance may be influenced by a country’s healthcare resources and/or benefit-to-cost ratios. The present study used recent patient records to examine uses of healthcare resources and their associated costs across 5 European countries in the ABC setting. The results demonstrated that total direct costs over the first 3 lines of therapy for HR^+^, HER2^−^ ABC in postmenopausal women were €10 000 to €14 000 lower if a hormone therapy-based regimen was used for 1 additional line of therapy versus switching to chemotherapy. The increase in direct costs for chemotherapy versus hormone therapy was also found in first-line and second-line treatments individually. Moreover, chemotherapy costs were increased despite longer duration of therapy in the cohort receiving hormone therapy.

The results of this study are supported by a study that found increased treatment costs associated with chemotherapy compared with hormone therapy in the ABC setting. A recent evaluation of total direct costs in the US for treating ABC reported that the monthly per-patient direct cost was lowest with systemic hormone therapy ($5303; *n* = 3187) compared with HER2-targeted therapy ($10 083; *n* = 711) or chemotherapy ($13 261; *n* = 2278) and was highest with no systemic therapy at all ($13 926; *n* = 1522) [13]. Until the present study, similar studies in Europe had not been performed.

Potential cost improvements may have been lost for patients who were eligible for and could have received benefit from hormone therapy in second line but who instead received chemotherapy. Accordingly, this study further examined the possible reasons for the increased cost associated with chemotherapy-based regimens. There were increased healthcare resource utilization costs for monitoring events, complementary therapies to manage side effects, and physician visits with chemotherapy-based regimens compared with hormone therapy. These findings are supported by a US study of 1444 women receiving chemotherapy for ABC, wherein healthcare resources other than the cost of chemotherapy comprised >50 % of the total costs: outpatient services accounted for 29 % of the total cost and medications other than chemotherapy accounted for 26 % [19]. In addition, patients receiving chemotherapy also had greater targeted therapy use compared with patients receiving hormone therapy in our study. Globally, the general use of targeted therapies will most likely increase as more of these agents are shown to provide clinical benefit and are approved. In the future, targeted therapies may also be used increasingly in combination with hormone therapy. Consequently, the total costs for hormone therapy-based therapy will increase. However, combinations with targeted agents may allow the extended use of lower-cost hormone therapy in patients who may derive clinical benefit, allowing a delay in switching to cytotoxic chemotherapy. In this study, the group of patients receiving targeted therapy in combination with hormone therapy was too small to be evaluated. We anticipate that a more in-depth review of these costs will become feasible in the future.

Another increased cost associated with chemotherapy-based versus hormone therapy-based regimens was indirect cost from lower work productivity, with a 3-fold lower proportion of patients working during second-line chemotherapy compared with hormone therapy. Overall, indirect costs associated with work status vary according to age. For example, a Swedish study stratified the total cost of all breast cancer cases in 2002 and reported higher indirect costs in breast cancer from sick leave, early retirement, and premature mortality (70 % of total) compared with direct costs [20]. However, the primary reason indirect costs dominated the total cost was because most of these breast cancer cases were in patients <65 years of age who were still in the workforce. Patients in the present study had a median age of 63 years; therefore, in theory, the working population accounted for ~50 % of the study’s total population, which would lessen the effect of indirect costs. A US study modeling the total costs specifically for ABC over 5 years (based on data from 2007) reported that lost work productivity accounted for only 21 % of the total cost for ABC [21]. The present study is the first to report a detailed assessment of work status over time stratified by treatment regimen in the ABC setting.

Limitations of this study are those primarily inherent to chart reviews. As with any chart review, there are limitations to the information available retrospectively that could have affected treatment decisions, such as accurate assessment of HER2 status. Although the inclusion criteria stated HER2^−^ disease, trastuzumab and lapatinib were used in a small percentage of patients. It is unclear whether these patients had confirmed HER2^−^ disease and HER2-targeted therapies were used because there were limited treatment options, the patients had unconfirmed HER2^−^ disease and HER2-targeted therapies were used as general practice, or the patients had participated in a past trial of HER2-targeted therapy that did not require documented HER2^+^ status at study entry. In some cases, the anti-HER2 therapy might have been used when the metastatic site was not able to be biopsied with the expectation that the tumor characteristics might have changed. Additionally, physicians may have based the treatment on results from the EGF30008 trial of lapatinib in combination with endocrine therapy [22].

In addition, accurate detailed information on the therapeutic regimens may be limited. These concerns were somewhat mitigated by having the treating physician complete the questionnaire using relatively recent patient charts. However, information that the treating physician may not be familiar with may be limiting, such as an accurate number of HCP visits for drug administration that can result in underestimation of utilization costs. In addition, HCP visits could have been underreported. In that case, HCP visit costs could be higher than the reported costs for the lines of therapy and cohorts wherein chemotherapy was used.

Another limitation to this study is the assumption that unit costs were the same within each country. Costs were calculated for each patient based on national costs in the country of the patient. However, local differences may exist that would introduce uncertainties into the difference between chemotherapy and hormone therapy costs. This study presents an average cost difference across the 5 European countries. Furthermore, standard medical practices are similar across the countries included in this study.

Finally, although the chart review covered treatments received from 2008 to 2012, only 2012 reference costs were used. However, the inflation rate in the European Union was ~8.1 % between 2008 and 2012, which is not considered to be a significant enough change to impact the resource utilization frequency/distribution [17].

## Conclusions

In this first study to evaluate the real-world experience in treating ABC in Europe, chemotherapy-based regimens appear to be associated with increased total direct costs compared with hormone therapy-based regimens. The results of the ongoing OPTIMA study in the United Kingdom are awaited to further aid in making the decision to initiate chemotherapy in patients with breast cancer and will also include a cost analysis [23]. Current international guidelines for the treatment of ABC recommend hormone therapy as long as patients are eligible (i.e., responsive and without symptomatic visceral metastases or need for rapid treatment control) [5, 8–10, 12], and this study supports these guidelines with regard to healthcare resource utilization, healthcare costs, and work productivity.

## Additional files

Additional file 1: Table S1. List of authorities the survey methodology was compliant with at the time of survey administration. (DOCX 16 kb)
Additional file 2: Figure S1. Survey questionnaire.
