# Supplementary material for: Patterns of resource utilization and cost for postmenopausal women with hormone-receptor–positive, human epidermal growth factor receptor-2–negative advanced breast cancer in Europe
Source: BMC Cancer. 2015 Oct 24;15:787. doi: 10.1186/s12885-015-1762-3 (PMC4619560; doi:10.1186/s12885-015-1762-3)
Supplement: Additional file 1: Table S1. — List of authorities the survey methodology was compliant with at the time of survey administration. (DOCX 16 kb) [file 12885_2015_1762_MOESM1_ESM.docx]

**Supplementary Table S1—List of authorities the survey methodology was compliant with at the time of survey administration**

| **Market Research Associations** | | |
| --- | --- | --- |
| ADETEM | L'association Nationale du Marketing | [www.adetem.org](http://www.adetem.org) |
| AMA | American Marketing Association | <http://www.marketingpower.com/> |
| AIMRI | Alliance of International Market Research Institutes | <http://www.aimri.net/> |
| AQR | Association for Qualitative Research | <http://www.aqr.org.uk/> |
| ESOMAR | European Society for Opinion and Marketing Research | <http://www.esomar.org/> |
| MRA+ CMOR | Market Research Association (merged with CMOR in November 2008) | <http://www.mra-net.org/> |
| Syntec | Syntec Etudes Marketing et Opinion | <http://www.syntec-etudes.com/Default_anglais.asp> |
| MRAgencies | Market Research Agencies | [www.MarketResearchAgencies.eu](http://www.MarketResearchAgencies.eu) |
| MRS | Market Research Society | <http://www.mrs.org.uk/> |
| CASRO | Council of American Survey Research Organizations | [www.casro.org](http://www.casro.org) |
| AQR | Association for Qualitative Research | <http://www.latrobe.edu.au/aqr/> |
| **Pharma Associations** | | |
| BHBIA | British Healthcare Business Intelligence Association | <http://www.bhbia.org.uk/> |
| EphMRA | European Pharmaceutical Marketing Research Association | <http://www.ephmra.org/main.asp?page=0> |
| PBIRG | Pharmaceutical Business Intelligence Group | <http://www.pbirg.com/new/home/default.asp> |
| PMRG | Pharmaceutical Marketing Research Group | <http://www.pmrg.org/> |
