# Supplementary material for: Patterns of resource utilization and cost for postmenopausal women with hormone-receptor–positive, human epidermal growth factor receptor-2–negative advanced breast cancer in Europe
Source: BMC Cancer. 2015 Oct 24;15:787. doi: 10.1186/s12885-015-1762-3 (PMC4619560; doi:10.1186/s12885-015-1762-3)
Supplement: Additional file 2: Figure S1. — Survey questionnaire. [file 12885_2015_1762_MOESM2_ESM.pdf]

Advanced Breast Cancer Chart Audit and Resource Utilization Questionnaire

GENERAL SCREENER QUESTIONS

S1. In which country do you practice?
S2. Please indicate in which region / hospital of your country you work when treating breast cancer patients.
S3. In what type of medical institution do you work when treating breast cancer patients?
S4. What is your primary medical specialty?
S5. Please indicate how long you have been in clinical practice post-residency or fellowship.
S6. Please indicate what percent of your professional time is spent treating patients.
S7. In the past 12 months, for how many breast cancer patients (new and returning) have you been the **primary** physician responsible for making **systemic** treatment decisions?

PATIENT CHART-SPECIFIC QUESTIONS

[[Section repeats for each patient chart being reviewed]]

A) INITIAL PRESENTATION AND DIAGNOSIS

A1. How old was the patient **at the time of diagnosis of advanced breast cancer**?
A2. What was this patient's menopausal status **at the time of diagnosis of advanced breast cancer**?
A3. In what year was this patient diagnosed with **advanced breast cancer**?
A4. Which option best describes the status of this patient **at the time of diagnosis of advanced breast cancer**? *[[Options include de novo advanced disease and recurrence during/after adjuvant therapy]]*
A5. Is there a history of breast or ovarian cancer in this patient's close family?
A6. Please indicate the *BRCA1* and *BRCA2* gene status for this patient.
A7. Did you order a **primary (breast)** tumour biopsy **at the time of diagnosis of advanced breast cancer**?
A8. Please specify this patient's tumour characteristics **at the time of diagnosis of advanced breast cancer**.
A9. Please indicate the location, extent, and molecular characteristics of this patient's metastatic sites **at the time of diagnosis of advanced breast cancer**.
A10. Please select all comorbidities that the patient had **at diagnosis of advanced breast cancer**.
A11. What was this patient's working status **at diagnosis of advanced breast cancer**?

B) DRUG THERAPIES BEFORE ADVANCED BREAST CANCER

B1. Please select all **drug** therapies that this patient had received in the **adjuvant setting.**

C) DRUG THERAPIES SINCE ADVANCED BREAST CANCER DIAGNOSIS

C1. How many **active** lines of therapy has this patient received **since she was diagnosed with advanced breast cancer**? Exclude best supportive care as a line of therapy. Please treat any maintenance therapy, if applicable, as a separate line of therapy.
C2. Please select the **number** of drugs that this patient received **during each line of therapy** after she was diagnosed with **advanced breast cancer**. Select all drugs administered during each individual line of therapy. Specify the duration for each therapy [in months].

D) FIRST LINE DRUG THERAPY AFTER ADVANCED BREAST CANCER DIAGNOSIS

[[This set of questions will repeat for each line of drug therapy depending on number of lines of treatment selected in C1; answer options include standard chemotherapy agents, hormonal therapy agents, and targeted agents with the option of entering investigational agents within each category.]]

D1. Please specify the drug compounds received during the **zth** line of therapy. Please specify the **average** dose per administration during the line of therapy, taking into account any dose reductions or increases. Please also specify the mode of administration of the drug and which setting the drug was predominantly administered.
D2. What was the main reason for choosing this regimen?
D3. Why did you choose this therapy over ------------? [[------------ refers to hormonal therapy if chemotherapy was the selected type of treatment, and vice versa]]
D4. By the time patient **started** the **zth** line of therapy, had there been a change in the extent, location or molecular characteristics of this patient's metastatic sites compared to the **start** of **(z–1)th** line of therapy?
D5. What was patient's performance status **at the start** of the **zth** line of therapy?
D6. What was patient's performance status **at the end** of the **zth** line of therapy?
D7. Please select all comorbidities that the patient had **at the start** of **zth** line of therapy.
D8. What type of tests had the patient received **during this line of therapy** to monitor disease progression and/or therapy-related side-effects?
D9. How many healthcare provider appointments and/or hospitalizations has this patient required **during this line of therapy**?
D10. For each hospitalisation event (inpatient hospital setting) during this line of therapy, please specify the main cause for hospitalisation, length of hospitalisation and the DRG / HRG code, if known.
D11. What type of **chemotherapy** side effects has the patient experienced **during** the **zth** line of therapy?
D12. What type of complementary treatments to prevent or treat **chemotherapy** side effects has the patient received **during zth** line of therapy?
D13. Please select the option that best describes this patient's working status during the majority of **zth** line of therapy.
D14. Please select the main reason for discontinuation (if applicable) of **zth** line of therapy and the switch to the next line of therapy.

E1. Would you like to share any additional comment with regards to this patient chart?

CONCLUDING QUESTIONS

*This question refers to **all** patient charts.*

F1. You specified that the patients received the following tests to monitor disease progression *[[list from D9]]*: Please specify the location for each monitoring test and the health care provider responsible for conducting the test.
